# Chemical entity recognition in patents by combining dictionary-based and statistical approaches

**DOI:** 10.1093/database/baw061

**Published:** 2016-05-02

**Authors:** Saber A. Akhondi, Ewoud Pons, Zubair Afzal, Herman van Haagen, Benedikt F.H. Becker, Kristina M. Hettne, Erik M. van Mulligen, Jan A. Kors

**Affiliations:** ^1^Department of Medical Informatics, Erasmus University Medical Center, PO Box 2040, 3000 CA Rotterdam; ^2^Department of Human Genetics, Leiden University Medical Center, PO Box 9600, 2300 RC Leiden, The Netherlands

## Abstract

We describe the development of a chemical entity recognition system and its application in the CHEMDNER-patent track of BioCreative 2015. This community challenge includes a Chemical Entity Mention in Patents (CEMP) recognition task and a Chemical Passage Detection (CPD) classification task. We addressed both tasks by an ensemble system that combines a dictionary-based approach with a statistical one. For this purpose the performance of several lexical resources was assessed using Peregrine, our open-source indexing engine. We combined our dictionary-based results on the patent corpus with the results of tmChem, a chemical recognizer using a conditional random field classifier. To improve the performance of tmChem, we utilized three additional features, viz. part-of-speech tags, lemmas and word-vector clusters. When evaluated on the training data, our final system obtained an *F*-score of 85.21% for the CEMP task, and an accuracy of 91.53% for the CPD task. On the test set, the best system ranked sixth among 21 teams for CEMP with an *F*-score of 86.82%, and second among nine teams for CPD with an accuracy of 94.23%. The differences in performance between the best ensemble system and the statistical system separately were small.

**Database URL**: http://biosemantics.org/chemdner-patents

## Introduction

Exploration of the chemical and biological space covered by patents is essential in the early stages of activities in the field of medicinal chemistry (1). Analyzing patents can help to understand compound prior art and to pinpoint alternative starting points for chemical research (2). Important tasks in patent analysis are the recognition of chemical names, the identification of chemical structure images, and the conversion of the extracted names and images into a structure-searchable form (3). Other types of entities in medicinal chemistry patents, such as genes and proteins, diseases, or particular numerical values, may also be relevant to extract and to relate to chemical entities (4). The extracted information is often compiled in structured databases that are easy to query and facilitate computational analysis.

Usually, patent information is manually extracted (5). This process is laborious and expensive due to the length of chemical patent texts, which may take hundreds of pages, and their complexity (mixture of scientific, technical and legal language, typographical errors, optical character recognition errors, etc.). These problems are aggravated by the sheer number of medicinal chemistry patents (1, 6). Automatic methods to recognize chemicals in patents can help to ease this process, but have proven to be elaborate and demanding (7, 8). One of the impediments is that very few large annotated gold-standard corpora for algorithm training and testing are available (9).

The automatic extraction of chemical and biological data from medicinal chemistry patents was addressed in the CHEMDNER-patents track of BioCreative V (10). The track was organized as a community challenge to stimulate the development and comparative assessment of chemical and biological entity recognizers, and consisted of three tasks: (i) Chemical Entity Mention in Patents (CEMP), focusing on chemical entity recognition in patents; (ii) Chemical Passage Detection (CPD), focusing on the classification of patent titles and abstracts according to whether they contain chemical entities; and (iii) Gene and Protein Related Object (GPRO), focusing on the recognition of gene and protein mentions in patents. Our team participated in the CEMP and CPD tasks.

Previous text-mining research mostly concentrated on chemical name recognition in scientific literature (4, 11). Recently, a large-scale patent resource, SureChEMBL (12), has become available, which contains compounds extracted from the full-text, images and attachments of patents, and provides comprehensive search capabilities. Chemical entity recognition is the first step in the SureChEMBL data extraction pipeline, but performance figures have not been presented as yet (12). A variety of systems to extract chemicals from Medline abstracts were developed and evaluated as part of the previous BioCreative IV CHEMDNER task (11). The top-ranking systems in that challenge used machine-learning techniques based on conditional random fields (CRFs) (11). However, some systems that combined dictionary-based and rule-based approaches also achieved competitive results (13, 14). For the current challenge, we combined a dictionary-based approach with a statistical, CRF-based approach, and investigated the performance of the ensemble system for the CEMP and CPD tasks on the CHEMDNER-patents data.

## Materials and methods

### Data

The CHEMDNER-patent corpus (10) was used for the development and evaluation of our system. The corpus comprises a training corpus of 14 000 manually annotated patent records (each record consisting of a title and an abstract), divided into a training set and a development set of 7000 records each, and a test set of 40 000 patent records, of which only 7000 were manually annotated. The annotation process and guidelines were largely similar to the ones used for the BioCreative IV CHEMDNER corpus, and have been described extensively (10, 15). Table 1 summarizes the number of annotated chemicals and chemical-related titles and abstracts. Only the annotations of the training and development sets were made available to the participants in the challenge. For evaluating the performance of their system on the test set, teams could submit up to five runs. To produce the evaluation results, we used the BioCreative evaluation software (www.biocreative.org/resources/biocreative-ii5/evaluation-library/) and focused on micro-averaged recall, precision and *F*-score to assess system performance for the CEMP task, and on sensitivity (=recall), specificity and accuracy for the CPD task. Given the number of true-positive (TP), false-positive (FP), false-negative (FN) and true-negative (TN) detections, these metrics were computed as follows: recall = TP/(TP + FN), precision = TP/(TP + FP), *F*-score = 2*precision*recall/(precision + recall), specificity = TN/(TN + FP) and accuracy =  (TP + TN)/(TP + FN + FP + TN). We also used the Markyt prediction analysis toolkit (www.markyt.org/biocreative/analysis) to visualize the results.

**Table 1.** Characteristics of the CHEMDNER patent corpus

	Training	Development	Test	Total
Patent records	7000	7000	7000	21 000
Manual chemical annotations	33 543	32 142	33 949	99 634
Unique chemical annotations	11 977	11 386	11 433	34 796
Chemical-related titles and abstracts	9152	8937	9270	27 359

### Dictionary-based approach

We used Peregrine, our open-source indexer (16), to analyze the performance of the different chemical dictionaries. Tokenization was done with a tokenizer previously developed by Hettne *et al.* (17). Term matching was carried out by partial case-sensitive matching: case-sensitive for abbreviations (defined as terms of which the majority of characters consists of capitals and digits), case-insensitive for all other terms.

#### Dictionaries

To construct our dictionaries, we selected seven well-known, publicly available chemical databases covering a wide range of compounds, namely: Chemical Entities of Biological Interest (ChEBI) (18), ChEMBL (19), DrugBank (20), the Human Metabolome Database (HMDB) (21), the NCGC Pharmaceutical Collection (NPC) (22), PubChem (23) and the Therapeutic Target Database (TTD) (24). For each database record, we gathered all chemical terms (available from possibly different record fields). Chemical terms were only extracted from records that had associated chemical structures in the form of MOL files (25). In the following, we briefly describe the databases and the fields from which identifiers were extracted.

ChEBI is concerned with molecular entities, focusing on small chemical compounds (18). It provides an ontological classification with parent and child relationships. We extracted data for all three-star (i.e. manually annotated) compounds from ChEBI SD files. This included synonyms, ChEBI names, brand names, International Nonproprietary Names (INNs) and International Union of Pure and Applied Chemistry (IUPAC) names.

ChEMBL contains information on drug-like bioactive compounds (19). In addition to literature-derived data, ChEMBL also contains Food and Drug Administration (FDA) approved drugs. The data available through ChEMBL have been manually extracted and standardized (26). Extracted fields include preferred names, synonyms, FDA alternative names, INNs, United States Adopted Names (USANs) and United States Pharmacopoeia (USP) names.

DrugBank provides information regarding drugs, including chemical, pharmacological and pharmaceutical data, and their targets (27). DrugBank data are curated by a curation team, which relies on primary literature sources. During production and maintenance, all synonyms and brand names within DrugBank are extensively reviewed and only the most common synonyms are kept (20). We extracted brand names, generic names, synonyms, Chemical Abstracts Service (CAS) numbers, and IUPAC names from the DrugBank SD files and DrugCards.

HMDB lists small-molecule metabolites found in the human body (21). The database links chemical, clinical, molecular-biology and biochemistry data. HMDB is both automatically and manually curated (21). All generic names, synonyms, CAS numbers and IUPAC names were extracted from the HMDB SD files and MetaboCards.

NPC provides information on clinically approved drugs from USA, Europe, Canada and Japan for high-throughput screening (22). We extracted preferred names and synonyms using the NPC browser 1.1.0.

PubChem provides information on the biological activity of small molecules (23). It consists of three different databases: a compound database, a substance database and a bioassay database. We extracted structures and all corresponding IUPAC identifiers and synonyms for a subset of compounds that had structure–activity relationships or other biological annotations. This subset of compounds was introduced by Muresan *et al.* (1) and is the same subset of PubChem compounds that we used in our previous study on chemical entity recognition (13). The PubChem compound database does not contain synonyms. This information is available through the PubChem substance database. The relations between PubChem substance identifiers (SIDs) and compound identifiers (CIDs), which have been created by PubChem through in-house chemical structure standardization (23), are specified in the ‘PubChem_CID_associations’ tag available in the downloadable structure data files. We used the relations between SIDs and CIDs to extract the synonyms from the substance database and assign them to the corresponding compounds.

TTD contains information about therapeutic protein and nucleic acid targets of drugs, corresponding pathways and targeted diseases (24). All trade names, drug names, CAS numbers and synonyms were extracted.

#### Dictionary construction and combination

For each database, a dictionary consisting of the extracted chemical terms was constructed. Each term was linked to one or possibly more (in case of ambiguity) compounds, represented by their MOL files. Dictionaries were combined by merging the identifiers of all compounds in the dictionaries. To determine which compounds in different dictionaries were the same, we used the same approach as in previous studies (28, 29). Briefly, we compared MOL files by converting them into InChI strings, which provide unique textual representations of the MOL files. Compounds with identical InChI strings were considered the same, and the corresponding identifiers were merged.

#### Term exclusion

To improve the precision of the dictionary-based approach, we applied an exclusion list of terms as previously described (13). Briefly, the list contains common English words, like ‘about’, ‘all’ and ‘make’, and ambiguous terms, such as ‘acid’, ‘crystal’ and ‘lead’. We expanded this list with exclusion terms mentioned in the annotation guidelines for the CEMP task.

We also removed terms that were false-positive detections in the training data, but only if the ratio of true-positive to false-positive detections was lower than 0.3. This threshold was heuristically set based on the training data in order to prevent erroneous removal of overall correctly recognized terms because of an occasional false-positive detection. When testing on the development set, exclusion ratios were calculated for all false-positive terms in the training set; when evaluating on the test set, ratios were computed for all false-positive terms in the combined training and development sets.

#### Term inclusion

We identified all missed terms (false negatives) in the training set and re-indexed the texts for these terms. Only those terms that, after re-indexing, did not result in false-positive detections in the training set or had an exclusion ratio larger than 0.5 were added to the dictionary. When evaluating on the test set, the combined training and development sets were used to collect the false negatives and to determine whether they should be included in the dictionary.

### Machine-learning approach

We used the tmChem chemical recognizer system (30), one of the best performing systems in the previous BioCreative CHEMDNER challenge (11). The tmChem system is an ensemble system that combines the output of two CRF-based systems. The first system is a modified version of the BANNER system (31), the second is based on the tmVar system (32), which employs CRF ++ libraries (https://taku910.github.io/crfpp/). Previous results of tmChem showed that the second system outperformed the first as well as the ensemble system (30). We therefore only used the second system.

#### Pre-processing

The tmChem system transliterates non-ASCII Unicode characters to a similar ASCII equivalent. As some non-ASCII Unicode characters were not handled (causing a system crash when encountered in text), we expanded the transliteration capacities as necessary. We also replaced a vertical bar enclosed by parentheses or brackets (e.g. [|]), because these combinations caused tmChem to crash as well.

#### Features

Our initial feature set consisted of all features extracted by tmChem, including stemmed words, prefixes and suffixes, character counts (digit, uppercase, lowercase), semantic affixes (such as trivial rings) and chemical elements (30).

Three additional types of features were determined and used to train tmChem: part-of-speech (POS) tags, lemmas and word-vector clusters. We used the BioC natural language processing pipeline (33) to generate POS tags with MaxentTagger (34) and lemmas with BioLemmatizer (35). Recent studies have shown that features based on clusters of word vectors can improve classification performance (36, 37). We used the word2vec tool (https://code.google.com/p/word2vec/) to generate clusters of word vectors. Word2vec employs K-means clustering. The number of the cluster to which a word belonged was taken as a feature.

We generated separate word clusters during the development phase and the test phase of the challenge. During development, the clusters were generated from the 14 000 titles and abstracts in the training and development sets. These data were extended with 200 full-text chemical patents that had been used in a previous study (9). We experimented with different numbers of clusters (*K* = 300, 500, 1000). For testing our final system, clusters were generated using all 54 000 records in the corpus plus the 200 full-text patents, with *K* = 1000.

### Post-processing

For the machine-learning approach, the tmChem post-processing steps were applied (30). These include enforcing tagging consistency (for each term that was found by the CRF at least twice within an abstract, any term mention in the abstract that the CRF had not identified was also tagged), abbreviation resolution (tagging corresponding abbreviations and long forms), boundary revision (adding or removing unbalanced brackets or parentheses) and finding chemical database identifiers (through regular expressions).

We experimented with different sets of dictionaries for the dictionary-based approach and different sets of features for the machine-learning approach. All terms recognized by the dictionary-based system or the statistical system were taken as the output of the final ensemble system.

### Text classification

For the CPD task (classification of patent titles and abstracts as chemical-related or not), we used a straightforward approach based on the output of the CEMP task. If our system recognized any chemical term in a text (title or abstract), the text was categorized as a chemical-related. Note that the title and abstract of each record were classified separately.

## Results

Table 2 shows the number of compounds and the number of unique identifiers in the chemical databases. Clearly, PubChem is by far the largest database. The number of identifiers that are shared between pairs of databases is shown in Table 3. Although PubChem contains >90% of the identifiers in ChEMBL, DrugBank and TTD, the other databases are much less well covered by PubChem. The majority of identifiers in DrugBank is covered by NPC and TTD, but the overlap between all other pairs of databases is relatively low.

**Table 2.** Number of compounds and unique identifiers in chemical databases

Database	No. of compounds	No. of identifiers
ChEBI	23 240	82 612
ChEMBL	22 245	28 411
DrugBank	6516	31 948
HMDB	40 199	228 907
NPC	14 666	128 153
PubChem	4 235 189	19 049 175
TTD	3196	121 744

**Table 3.** Number of unique identifiers that overlap between pairs of chemical databases

Database	ChEBI	ChEMBL	DrugBank	HMDB	NPC	PubChem
ChEMBL	1209 (4.3)					
DrugBank	2444 (7.6)	3931 (13.8)				
HMDB	4885 (5.9)	2293 (8.1)	5946 (18.6)			
NPC	3406 (4.1)	6508 (22.9)	23 865 (74.7)	7444 (5.8)		
PubChem	45 021 (54.5)	26 251 (92.4)	28 943 (90.6)	52 533 (22.9)	69 873 (54.5)	
TTD	4481 (5.4)	4507 (15.9)	18 028 (56.4)	6503 (5.3)	23 901 (19.6)	119 819 (98.4)

The percentage coverage of the identifiers in the smallest sized database of each pair is given in parentheses.

Table 4 shows the performance of the dictionary-based approach on the development set, with and without use of the list of exclusion terms. Use of the exclusion list gives a substantial precision improvement for most dictionaries. The PubChem dictionary demonstrates the highest recall among the individual dictionaries, which may be explained by the large size of the PubChem dictionary and the fact that it contains the majority of terms from the other dictionaries. The dictionaries from ChEMBL and DrugBank had the highest precision, which is likely due to the fact that these databases are highly curated. The low recall of the dictionaries can be explained by their low coverage of systematic names and chemical family names. Of the 9194 systematic names that were annotated in the development corpus, recognition rates ranged from 7.5% for TTD to 53.8% for PubChem (median 31.0%). For family names, which form the largest annotation group (*n* = 11 710), recognition rate varied between 3.3% and 20.4% (median 9.1%).

**Table 4.** Performance of different dictionaries and dictionary combinations with and without removal of exclusion terms

	Without exclusion			With exclusion		
Dictionary	Precision	Recall	*F*-score	Precision	Recall	*F*-score
ChEBI	56.51	29.47	38.74	78.87	28.42	41.79
ChEMBL	84.53	20.46	32.94	85.11	19.87	32.22
DrugBank	68.20	17.28	27.58	85.15	16.89	28.19
HMDB	66.11	29.38	40.68	79.59	28.19	41.63
NPC	30.90	44.85	36.59	55.23	30.61	39.39
TTD	66.89	14.07	23.24	80.90	13.89	23.71
PubChem	34.30	47.11	39.69	67.03	45.64	54.30
All combined	30.85	50.32	38.25	53.66	48.59	51.00
ChEBI–HMDB	55.46	36.98	44.37	78.12	35.45	48.77
ChEMBL–DrugBank	70.51	23.94	35.74	83.02	23.16	36.21

Table 4 also shows the performance of several combinations of dictionaries. As to be expected, the combination of all dictionaries after term exclusion has the highest recall (49%), but the lowest precision (54%). The combination of dictionaries from ChEBI and HMDB, which we used in the previous BioCreative CHEMDNER task (13), gave a recall of 35% and a precision of 78%. The combination of ChEMBL and DrugBank resulted in the highest precision (83%).

Table 5 shows the incremental performance of the ensemble system trained on the training corpus and evaluated on the development corpus, when different feature sets and term-processing steps were added. We only present dictionary-based results for the combination of ChEMBL and DrugBank as this combination produced the highest *F*-score on the training data when combined with the CRF. For the CEMP task, all incremental steps improved the *F*-score, except when terms that were missed in the training set were included in the dictionary. The best ensemble system attained an *F*-score of 85.21% with a precision of 84.88% and a recall of 85.55%. For the CPD task, the system that comprised all processing steps, including the addition of missed terms, achieved the best performance with an accuracy of 91.84% (sensitivity 97.00%, specificity 82.74%).

**Table 5.** Performance of the ensemble system trained on the training set and tested on the development set

	CEMP task			CPD task		
System	Precision	Recall	*F*-score	Sensitivity	Specificity	Accuracy
Dictionary-based (ChEMBL-DrugBank)	70.51	23.94	35.74	50.63	88.41	64.29
+ Exclusion list	83.02	23.16	36.21	44.29	94.37	62.40
+ Term removal (exclusion ratio 0.3)	88.85	23.09	36.65	42.14	97.12	62.02
+ CRF original features	84.96	83.83	84.39	95.11	85.33	91.57
+ Post-processing (CRF)	84.50	84.91	84.70	95.39	85.01	91.64
+ POS and lemmatization features	84.72	85.09	84.90	95.40	85.25	91.73
+ Word-vector cluster features	84.88	85.55	85.21	95.31	84.87	91.54
+ Missed terms (exclusion ratio 0.5)	75.88	88.63	81.76	97.00	82.74	91.84

When we only used the CRF-based system (trained on all features) to process the development set, we obtained an *F*-score of 84.78% (precision 86.14%, recall 83.47%) on the CEMP task, and an accuracy of 90.96% (sensitivity 94.23%, specificity 85.19%) on the CPD task.

Table 6 shows the performance for both tasks on the test set. We submitted runs of the ensemble systems with and without the addition of missed terms. For comparison, we also submitted a run for the statistical system alone (including all features and post-processing).

**Table 6.** Performance of different systems on the test set

	CEMP task			CPD task		
System	Precision	Recall	*F*-score	Sensitivity	Specificity	Accuracy
Statistical	86.83	86.81	86.82	96.13	88.67	93.61
Statistical + dictionary without missed terms	84.92	88.25	86.55	97.00	87.91	93.93
Statistical + dictionary with missed terms	77.76	90.84	83.79	98.03	86.79	94.23

For the CEMP task, the statistical system performed best (*F*-score 86.82%), slightly better than the ensemble system without the addition of missed terms (*F*-score 86.55%). For CPD, the ensemble system with missed terms reached the best performance (accuracy 94.23%), slightly better again than the system without missed terms (93.93%). Our best systems ranked sixth among 21 participating teams for the CEMP task, and second among nine teams for the CPD task.

## Discussion

We investigated the combination of dictionary-based and statistical approaches for chemical entity recognition in patents. Our results show that the recall of the chemical dictionaries on the CEMP task is low, and even a combination of all dictionaries gives a recall and precision of only around 50%. The low recall can be explained by the fact that many systematic chemical terms and chemical family names were lacking in our lexical resources. Meanwhile, the machine-learning approach yielded a much higher precision and recall (86% and 83%, respectively). In order to maintain the high precision of the ensemble system, we used the dictionary combination with the highest precision (ChEMBL and DrugBank). For the CEMP task, this supplied us with a system that slightly improved machine-learning performance on the development set, but not on the test set. Thus, there was no performance gain for this task by the use of a combined dictionary-based and statistical approach over a statistical approach alone. For the CPD task, the ensemble system performed better than the statistical system alone, both on the development set and on the test set. This may be explained by the 1.9 percentage point higher sensitivity of the ensemble system, in combination with a similar decrease in specificity. As the majority of titles and abstracts in the development and test sets are chemical-related (see Table 2), sensitivity weighs more heavily than specificity in the accuracy. For both tasks, our results on the test set were better than those on the development set, indicating that overtraining did not occur.

Contrary to our expectation, the inclusion of false-negative terms in the dictionary decreased the performance for the CEMP task, both on the development set and on the test set. This may partly be explained by tokenization issues that split chemical terms in multiple parts. Some of these parts were then erroneously matched with the newly added dictionary terms, resulting in a drop in precision. For the CPD task, the increase in sensitivity more than compensated for the decrease in specificity, yielding a slightly improved accuracy of the ensemble system using the missed terms.

Although furnishing structure information about the recognized chemicals was not part of the challenge, this information is often important in practical applications. We are able to readily associate dictionary terms with structures because we only extracted terms from chemical records with structure information. Of the chemical terms in the development set, 23% is found by the dictionary-based approach and can be linked to structures. For the machine-learning approach, the mapping of recognized terms to structures is less straightforward, but part of these terms will consist of systematic chemical identifiers. These can also be converted into chemical structures using chemical naming conversion software (28, 29).

Considering that annotated patent corpora are scarce, the CHEMDNER corpus of annotated patent titles and abstracts is a highly valuable and important resource for further development and comparative assessment of algorithms. Recently, we have reported on the creation of another corpus of 200 annotated full-text patents, which is publicly available (9). We plan to use this corpus to evaluate and possibly improve the performance of our systems on full-text patents.

## Funding

AstraZeneca to S.A.A.; European Union Seventh Framework Programme (Grant Agreement No. 305444) (RD-Connect; HEALTH.2012.2.1.1-1-C) to K.M.H.

*Conflict of interest*. None declared.
